# Evidence for marine sedimentary rocks in Utopia Planitia: Zhurong rover observations

**DOI:** 10.1093/nsr/nwad137

**Published:** 2023-05-18

**Authors:** Long Xiao, Jun Huang, Timothy Kusky, James W Head, Jiannan Zhao, Jiang Wang, Le Wang, Wenchao Yu, Yutong Shi, Bo Wu, Yuqi Qian, Qian Huang, Xiao Xiao

**Affiliations:** State Key Laboratory of Geological Processes and Mineral Resources, Planetary Science Institute, School of Earth Sciences, China University of Geosciences, Wuhan 430074, China; Chinese Academy of Sciences Center for Excellence in Comparative Planetology, Hefei 230026, China; State Key Laboratory of Geological Processes and Mineral Resources, Planetary Science Institute, School of Earth Sciences, China University of Geosciences, Wuhan 430074, China; Chinese Academy of Sciences Center for Excellence in Comparative Planetology, Hefei 230026, China; State Key Laboratory of Geological Processes and Mineral Resources, Center for Global Tectonics, School of Earth Sciences, China University of Geosciences, Wuhan 430074, China; Badong National Observatory and Research Station for Geohazards, China University of Geosciences, Wuhan 430074, China; Department of Earth, Environmental and Planetary Sciences, Brown University, Providence, RI 02912, USA; Key Laboratory of Geological Survey and Evaluation of Ministry of Education, China University of Geosciences, Wuhan 430074, China; State Key Laboratory of Geological Processes and Mineral Resources, Planetary Science Institute, School of Earth Sciences, China University of Geosciences, Wuhan 430074, China; State Key Laboratory of Geological Processes and Mineral Resources, Planetary Science Institute, School of Earth Sciences, China University of Geosciences, Wuhan 430074, China; State Key Laboratory of Geological Processes and Mineral Resources, Planetary Science Institute, School of Earth Sciences, China University of Geosciences, Wuhan 430074, China; State Key Laboratory of Geological Processes and Mineral Resources, Planetary Science Institute, School of Earth Sciences, China University of Geosciences, Wuhan 430074, China; Planetary Remote Sensing Laboratory, Department of Land Surveying and Geo-Informatics, The Hong Kong Polytechnic University, Hong Kong100872, China; State Key Laboratory of Geological Processes and Mineral Resources, Planetary Science Institute, School of Earth Sciences, China University of Geosciences, Wuhan 430074, China; Hubei Subsurface Multi-scale Imaging Key Laboratory, Institute of Geophysics and Geomatics, China University of Geosciences, Wuhan 430074, China; State Key Laboratory of Geological Processes and Mineral Resources, Planetary Science Institute, School of Earth Sciences, China University of Geosciences, Wuhan 430074, China

**Keywords:** ancient ocean, sedimentary rocks, Zhurong, Tianwen-1, Mars

## Abstract

Decades of research using remotely sensed data have extracted evidence for the presence of an ocean in the northern lowlands of Mars in the Hesperian (∼3.3 Ga), but these claims have remained controversial due to the lack of *in situ* analysis of the associated geologic unit, the Vastitas Borealis Formation (VBF). The Tianwen-1/Zhurong rover was targeted to land within the VBF near its southern margin and has traversed almost 2 km southward toward the interpreted shoreline. We report here on the first *in situ* analysis of the VBF that reveals sedimentary structures and features in surface rocks that suggest that the VBF was deposited in a marine environment, providing direct support for the existence of an ancient (Hesperian) ocean on Mars.

## Teaser

Whether or not an ocean ever existed on Mars has been debated for decades. China's Zhurong rover has recently found ocean sedimentary rocks for the first time in the Utopia Planitia of Mars, which supports that there was an ancient ocean in Martian northern lowland.

## INTRODUCTION

**Figure 1.**
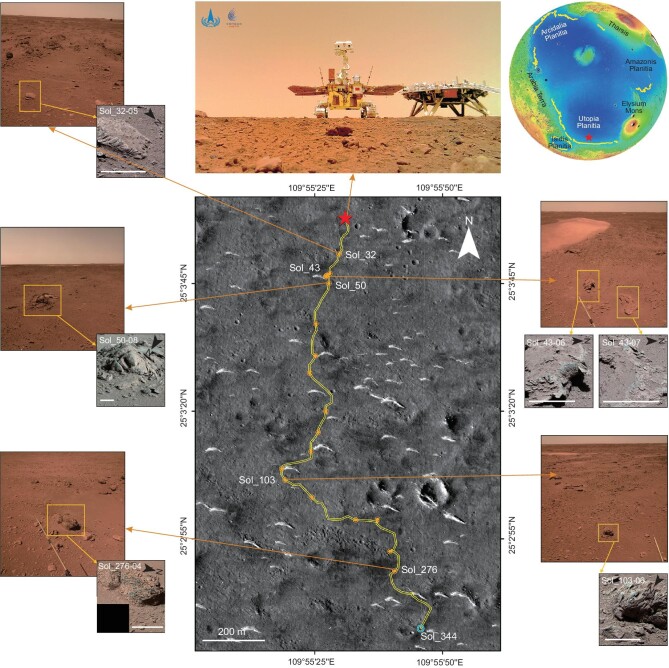
The traverse of the Zhurong rover and key observations (Sol 31, Sol 42, Sol 50, Sol 102 and Sol 274) of NaTeCam and (Sol 32, Sol 43, Sol 50, Sol 103 and Sol 276) MSCam as of 30 March 2022 (Sol_344). The star marks the landing site, where an image of the rover and lander was taken by a separate camera on the ground. Color-coded MOLA Mission Experiment Gridded Data Record with polar stereo projection (centered at 60°N, 120°E) shows the regional topographic characteristics with the star indicating the landing site in southern Utopia Planitia. Dots in the middle figure indicate the waypoints with both MSCam and NaTeCam observations. The line connecting these dots is an approximation of the traverse using the locations of the NaTeCam observations. The scales in all the MSCam images are 15 cm. The background image of the traverse is a mosaic of Tianwen-1 HiRIC images (HX1_GRAS_HIRIC_DIM_0.7_0004_251515N1095850E_A). The upper right Mars global topography figure is based on MOLA data and the curved line inside indicates the Deuteronilus shoreline [18].

The search for water habitats potentially conducive to life on early Mars has led to the recognition of widespread fluvial valley networks [1], open-basin lakes [2], closed-basin lakes [3], large-scale fluvial outflow channels [4], deltas (e.g. [5–7]) and alluvial fans (e.g [8]). In addition, the presence of a former ocean has been suggested by observations and interpretations of extensive deposition on the northern plains exposed in impact craters [9], isotopic studies of the Martian hydrosphere [10], analysis of base-level controls of progressive channel erosion [11] and radar properties of the northern plains [12]. Most dramatic in scale of these candidate aqueous environments are ocean-scale bodies [13] of water hypothesized to have occupied the northern lowlands of Mars in the Noachian (∼4.1–3.7 Ga) and Hesperian (∼3.7–3.0 Ga) eras of early Mars history [14]. However, degradation and Hesperian volcanic resurfacing, glacial, periglacial and mud volcanism processes in the northern lowlands [15] have obscured the features interpreted to be associated with the proposed Late-Noachian ocean (e.g. Arabia level [16]). The younger of these proposed shorelines (Late Hesperian; the Deuteronilus level [17] (Fig. 1)) is associated with the circum-Chryse region outflow channels [4], the sites of huge mega-floods of water, clearly emptying into the northern lowlands [13]. Analysis of the margins of the northern lowlands has revealed a curvilinear boundary interpreted as a former shoreline of this ocean. This interpretation is based on analysis of a zone of morphological change, approximately surrounding and paralleling the transition from the northern plains to the southern highlands of Mars. This zone extends for thousands of kilometers apparently preserving the shoreline of a huge ancient ocean produced by catastrophic floods reaching the northern plains through the outflow channels [18,19]. Geological mapping of the northern plains of Mars [20] has documented the presence of a lowlands-wide geological unit, the Vastitas Borealis Formation (VBF), whose Late-Hesperian age is contemporaneous with the fluvial outflow channels, and whose outer contact closely coincides with the features interpreted as a shoreline [18]. These characteristics and relationships have led many to interpret the VBF as the sedimentary deposit of an ancient oceanic body of water probably formed from massive influxes of water discharges from the outflow channels and other fluvial pathways, plus groundwater flows [13,21]. These outflow channels are thought to have been formed by cracking of the crust and cryosphere by dikes and fractures and the catastrophic release of pressurized groundwater [4]. In addition, evidence has been presented for distinctive deposits that are interpreted to represent the formation of at least two megatsunami, probably resulting from impacts into such an oceanic body of water [22–25] and separated by a major regression phase [24,25]. Recent scientific approaches to understanding the paleoclimate of ancient Mars have been dominated by computer modeling that applies well-verified physical laws to simulating past conditions on the planet (e.g. [26–30]). Many of these models (e.g. [29]) suggest that the residence time of a Hesperian ocean might be relatively short geologically, although other models (e.g. [27,28]) produce different results. However, almost all aspects of the proposed northern lowland oceans [31,32] and their shorelines [18,33,34] remain controversial.

An early synthesis and analysis of evidence for a northern lowland ocean [21] pointed out that the most compelling evidence for a Hesperian ocean was the presence, distribution, contacts and regional characteristics of the VBF, and recommended more detailed *in situ* study of this unit. However, the nature of the VBF, its internal structure and its manner and environment of formation have remained unknown. Direct evidence is required to examine whether the VBF is a residual sedimentary deposit representing an oceanic, shallow marine or coastal shoreline-related environment, or a mantling unit emplaced by spin-axis/orbital variations, or an aeolian deposit representing reworked earlier units formed during the last 3 billion years of Mars history.

On 15 May 2021, China's Tianwen-1/Zhurong rover successfully landed in the VBF in the northern lowlands of Mars (southern Utopia Planitia, 109.925°E, 25.066°N) [35,36] ∼500 m below and ∼282 km north of the southern VBF contact and proposed Deuteronilus shoreline [18] (Fig. 1 and Supplementary Fig. S1). Since its landing, it has been engaged in an exploration traverse southward toward the proposed shoreline, exploring potential surface exposures of the VBF and rocks excavated from the interior of the VBF by subsequent impacts, and the subsurface structure of the landing area. In the last 12 months, the Zhurong rover has travelled ∼1921 m, pausing on 18 May 2022 for winter dormancy. The area traversed is relatively flat with local elevation changes of ∼5 m (Fig. 1 and Supplementary Fig. S2). From an orbital perspective, the region is relatively dusty, inhibiting successful orbital spectrometer mineralogical determination [37]. The Zhurong rover, however, can make detailed *in situ* observations of surface rocks using different imaging and analysis systems, including (i) Navigation and Terrain Cameras (NaTeCam) [38], (ii) a Multispectral Camera (MSCam) [39] and (iii) the Micro-imaging Camera [40]. The NaTeCam has acquired 106 groups of panoramic images, revealing many boulders and blocks in the vicinity of the Zhurong traverse (Supplementary Figs S3–S5).

## RESULTS AND INTERPRETATIONS

The MSCam has documented 23 rocks. Most of them show laminations and are covered with varying degrees of dust on their surface. Five boulders and blocks were chosen for analysis in this study using the following selection criteria: (i) not severely modified by wind erosion, later alteration, including impact induced fracturing, and (ii) large enough to show the variations in the observed layers. Detailed morphological observations and analyses of characteristic features and internal structures are presented; these data form the basis for interpretation of their environment of origin. These rocks, several centimeters to several tens of centimeters in dimension, are scattered with various orientations (Fig. 1 and Supplementary Figs S3–S8), probably as a result of being ejected from the subsurface by nearby impact events. These exposed rocks are variably covered by wind-blown debris on the leeward surfaces and scoured by wind abrasion on the windward faces, forming broadly faceted surfaces as commonly seen on terrestrial ventifacts, whereas some pitted surfaces have been interpreted to be related to brine dissolution [35]. The locally well-etched faces preferentially highlight the presence of features interpreted to be primary sedimentary structures, indicating that they are all sedimentary rocks.

These observations reveal a wide range of sedimentary structures of the VBF, including bedding and inclined bedding features, documented along the almost 2-km-long traverse from the landing site in the VBF toward the proposed Deuteronilus level shoreline. In the following section, we describe the series of rocks excavated from the VBF by post-VBF impacts, outlining their internal structures and comparing them to features seen in different terrestrial [41] and known Martian environments [42,43] in order to assess their modes of origin and the origin of the VBF. Since these observations are all from the vicinity of the Zhurong rover traverse, we use the informal term ‘Zhurong Member of the VBF’ for the rocks described herein.

**Figure 2.**
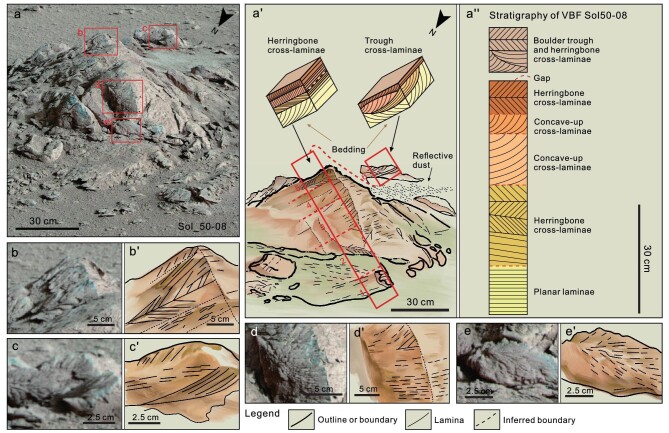
A boulder (Sol_50–8) showing typical shallow marine sedimentary structures of the Zhurong Member. Block diagrams show interpreted sedimentary structures. The boulder is ∼110 cm across and preserves a series of left-dipping planar surfaces separating 2- to 10-cm-thick tabular units interpreted to be primary sedimentary bedding and is generally consistent with that on the surrounding ground. The beds have internal laminations that differ from bed to bed. These features are used to interpret the primary depositional environment. Note that the boulder is locally sculpted by wind, in places covered by wind-blown dust or sand, and possibly affected by chemical weathering or cementation [45]. (a″) Stratigraphic section drawn perpendicular to the bedding along the front face of the outcrop, and placing boulder c on top, as it appears to strike with a small missing gap. The section is divided into six units of planar, herringbone, convex-up and trough cross-laminated layers, suggestive of deposition by bidirectional subaqueous currents. Details of features from the boxes on panel (a). (b) Features interpreted as herringbone cross-laminae formed by bipolar current directions, typical of alternating currents. (c) Features interpreted as trough cross-lamina from a separate boulder in the background, representing bipolar current directions, overlain by planar cross-laminated sand. The boulder is possibly out of place or even upside down, but appears to have similar orientation as the main boulder. (d) Features interpreted as planar bedding visible in 3D around a corner in the boulder, overlain by a concave cross-laminated unit. (e) Features interpreted as herringbone cross-lamina, strongly etched by wind, forming a pseudo-fan shape.

Sol_50–8 (Fig. 2) is an angular boulder ∼1 m across. Observed features include a series of ∼5- to 8-cm-thick left-dipping planes interpreted as bedding surfaces of sedimentary rocks (Fig. 2a′). These bedding surfaces bound internal cross-laminated layers that dip both left and right at ∼30°, and locally up to ∼42° from the main bedding, and are most prominently visible along the top of the exposure (Fig. 2b), the lower right corner (Fig. 2d and e) and on a block (in box Fig. 2c) behind the main boulder. Most of the cross-laminations are planar (Fig. 2d) but some (such as in the middle of the block) display curved concave-up cross-laminae that merge asymptotically with the underlying bedding surface (Fig. 2b–e). More rarely, the cross-laminations are slightly convex-up, as in the middle of Fig. 2b, and merge with the presumed upper surface, which is present in some trough cross-laminated deposits on Earth [44].

The sedimentary structures of the composite section from the main boulder and boulder **c** are shown in Fig. 2a″. In boulder c (Fig. 2c), the thicknesses of the lamina-sets are ∼2.5 cm, indicating that the height of the ripples was several centimeters. The size of the sedimentary structures suggests that the rock is composed of silt and fine-to-medium-grained sand particles (Supplementary Table S1). The cross-laminations include herringbone and trough forms (Fig. 2b, c and e), which differ from typical lacustrine or aeolian deposits on Earth [42,44] or Mars (e.g. Gale and Jezero crater basins) [43], and characterize shoreline environments with bidirectional current directions on Earth [46]. Some aeolian processes may produce slightly similar bidirectional deposits [47] but they are different from those described here in that in aeolian ‘bidirectional’ layers, there should be a mud layer interbedded between two sand layers that contain the oppositely dipping lamina. Thus, these planar and cross-laminations are interpreted to indicate deposition by an alternating aqueous current, and on Earth these are typical of tidal or foreshore environments [41] (Supplementary Fig. S9 and Supplementary Table S1).

**Figure 3.**
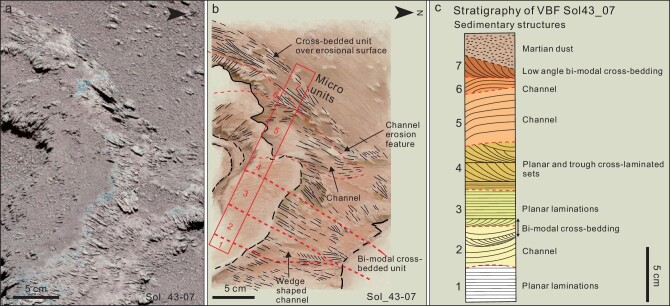
Multispectral Camera (MSCam) images show a variety of submarine environment depositional structures on the Block Sol 43–07 (a). The rocky surface shows seven main stratigraphic micro-units including, from base to top, (1) a planar laminated unit, (2) cross-bedded channel deposit, (3) a horizontal and planar cross-laminated unit with bidirectional dips on cross-laminations, (4) a planar and trough cross-bedded unit, (5) and (6) channel deposit with steep point-bar-like laminations and (7) low-angle cross-laminated unit. Panels (b) and (c) show a measured and interpreted schematic stratigraphic column of this small section of the VBF.

Boulder Sol_43–07 (Fig. 3) is ∼20 × 60 cm in dimension and exposes a series of beds and laminae interpreted to consist of alternating lens-shaped channels with internal cross-laminations and others oriented parallel to the channel, which are common in fluvial, tidal and related aqueous channel environments on Earth [48,49]. On the basis of the size of the cross-lamina (Supplementary Table S1) and micro-images in Supplementary Fig. S8, the speculative grain size of these sedimentary structures is made of coarse silt to very fine sand-sized particles. On the line drawing, we divide this outcrop into seven different micro-units that have slightly different characteristics (Fig. 3b). Some are planar tabular bedding units and three (Micro-units 2, 5 and 6, Fig. 3b and c) are small (5–15 cm) lenticular units that are interpreted to be small channels, each capped by several-centimeter-thick cross-laminated units that suggest bidirectional current flow. Overall, the interpreted channels and bidirectional currents in the capping units are not seen in aeolian environments and are similar to those seen in terrestrial low-energy shallow marine environments, and are distinctive from fluvial environments [43]. For most aeolian and fluvial deposits, sedimentary structures indicate a unidirectional mode showing the movement direction of the wind. However, certain sections cut obliquely across the transport direction of large-scale linear aeolian dunes sometimes show similar bidirectional patterns as in the column, but are significantly different in scale and morphology from the bidirectional deposit in the marine environment (Supplementary Fig. S9 and description). In Fig. 3c, we present an outcrop-scale stratigraphic section through boulder Sol_43_07 that shows the small-scale lenticular channels and bi-modal paleocurrent directions inferred from oppositely dipping cross-lamina. This stratigraphic section of the Zhurong Member, albeit a minor part of the whole VBF, clearly shows features remarkably distinct from aeolian and fluvial environments, and also different from the disorganized patterns of mud volcanoes, which were present in places on early Mars [50–52], but similar to low-energy subaqueous environments on Earth [49].

Block Sol_32–05 is a tabular block ∼20 cm long and 10 cm across (Supplementary Fig. S6). It displays an upper bright layer and a lower darker layer, interpreted as primary bedding. Grain-size analysis suggests that the upper layer is sandy and the lower layer is silt to mud (Supplementary Table S1, here we only provide a speculative grain size). Both beds show internal layering at angles to the main bedding. The most prominent are concave-up to planar laminations in the upper layer that dip to the left and converge on a plane (lower bedding surface) parallel to the upper surface of the block (Supplementary Fig. S6a and a′). We interpret these oblique layers as compound cross-laminations [53,54] in which the main layer consists of several internal bedforms that are themselves cross-laminated. Below this plane, the rock is darker and more recessed in the block than the upper part, indicating that this part is less resistant to the weathering than the prominent upper layer. Differential weathering is most likely due to the grain-size variation in the different layers, with recessed parts being finer-grained, with fainter laminations that dip to the right. Together, these sets of cross-laminations, with different grain sizes, suggest deposition by bidirectional currents of different strengths, which differs from aeolian deposits by virtue of the difference in grain size of the stacked layers [46]. The upper bed, displaying prominent compound cross-laminae, including concave-up cross-laminations each ∼2 cm long, is suggestive of compound cross-strata consisting of several descending sets of trough cross-laminations (Supplementary Fig. S6b and d). These are morphologically the same type as those typical of deposition/accretion along the downflow sides of migrating compound bedforms [41]. The boundary between the upper and lower beds shows lenticular features with characteristics of both the upper, coarser-grained layer and the lower, finer-grained layer (Supplementary Fig. S6c and c′). Some of the discontinuous features are inclined and some are parallel to the bedding surface, similar to lenticular and flaser-type bedding and mud drapes seen in mixed mud and sand deposits on Earth [49]. The cross-laminations in the lower bed are planar in cross section, dipping uniformly to the right (Supplementary Fig. S6a′). Together, these two beds with oppositely dipping cross-laminations indicate bipolar depositional currents. Furthermore, in terrestrial shallow submarine environments, such a thicker upper coarser-grained layer overlying a finer-grained lower layer (panels in Supplementary Fig. S6a and a′) has been interpreted to indicate a stronger flood tide, following a lower-energy ebb tide (Supplementary Fig. S9c).

Boulder Sol_43–06 (Supplementary Fig. S7) is ∼50 cm across. It displays about six left-dipping 1- to 3-cm-thick relatively light and dark parallel layers, interpreted as sedimentary beds of different grain size or composition. Grain-size analysis (Supplementary Table S1) suggests that these are dominantly fine-to-medium-grained sands. The prominent left- and right-dipping cross-laminations (Supplementary Fig. S7a and a′) features are not typically seen in aeolian or fluvial environments on Mars [43]. The top of the boulder is partly covered by dust but traces of the cross-laminations protrude through the dusty layer, exposing curved traces suggestive of trough cross-beds (see interpretation in lower part of panel in Supplementary Fig. S7a′). Wind erosion has formed small grooves on the sides of the boulder (color lines on panel in Supplementary Fig. S7a′) and may have enhanced the cross-laminations by etching. Two relatively bright areas on the right side of the rock may be either favorably oriented to reflect additional light or may be larger atypical cobble-sized fragments in the clastic sedimentary layers. Supplementary Figs S7b and 7c show enlarged sections of this block with line sketches; these appear consistent with them being either artifacts of the lighting and weathering, or primary sedimentary features. If they are larger sedimentary fragments, then they could be similar to residual blocks that are ripped up by currents in tidal systems on Earth or they could be pre-depositional ventifacts or erosional lags due to the transient exposure, and subsequently deposited with surrounding cross-laminated sandy layers [55,56].

Boulder Sol_103–6 consists of two main wind-sculpted blocks, 30 cm and >70 cm across (Supplementary Fig. S8a and b), consisting of fine-to-medium sand to mud-sized particles (Supplementary Table S1). Despite strong erosion by wind of these ventifacts, the larger one preserves several nearly parallel layers interpreted to be sedimentary in origin. Two of these (left and in the center of the boulder) have internal parallel to slightly oblique laminations (labeled with bedding symbols) whereas intervening layers show convex cross-laminations that merge with the underlying parallel laminated layers. In the large boulder, both layers with these laminations are similar to those in terrestrial marine environments influenced by currents. The small boulder in the foreground shows a relatively bright upper section (coarse-grained sand-sized particles) over a darker lower part (finer-grained mud particles). The upper part shows numerous prominent cross-laminations dipping at 30° to the left whereas the lower darker unit consists of several thinner finer-grained layers (beds) that have fainter and thinner flaser-like bedding consisting of small lenses of sand and mud, and cross-laminations that dip to the right. In the micro-images of the upper part of this boulder (Supplementary Fig. S8c and d), the scale of the lamina-set is ∼5 mm and the cross-lamina can be clearly observed in both the weathered surface (Supplementary Fig. S8c) and the fresh surface (Supplementary Fig. S8d), where the latter was formed due to the laser abrasion of the Mars Surface Composition Detector (MarSCoDe) instrument of the rover. Since the laser abrader removed the surface layer visible in (c) for producing the image in (d), we can show that the laminations are primary sedimentary features and not produced by wind abrasion. Together these appear to form an asymmetric herringbone-like cross-lamination set, which is indicative of bipolar current directions, with the flow to the left being stronger than the flow to the right. On Earth, the combination of (i) curved and planar cross-beds, (ii) herringbone cross-lamination and (iii) thin flaser bedding is typical of deposition in bidirectional tidal-influenced foreshore settings [41,55,57,58] and this assemblage is distinct from those formed in fluvial and aeolian environments (Supplementary Fig. S9a and b).

## DISCUSSION

Taken together, the boulders and blocks of the Zhurong Member of the VBF generally preserve parallel-tabular bedding with local lenticular bedforms, with a variety of interpreted grain sizes and sedimentary structures dominated by various types of cross-laminations, less-common lenticular and flaser bedding and small-scale channel structures. The cross-laminations and cosets dip in two opposite directions indicating bidirectional paleocurrent orientations and, since the thickness and grain sizes (Supplementary Fig. S9c) are systematically larger on sets dipping one way and not the other, the depositing paleocurrents can be inferred to have been alternatively weak and strong. Bidirectional current orientations are characteristically formed by the regularly opposite variation of tidal current directions and this current pattern is not commonly present in fluvial environments, but is common in the terrestrial shallow marine environment.

Bidirectional current orientations are characteristically formed by regular opposite directions of tidal currents in terrestrial shallow marine environments and uncommonly in fluvial environments. Although aeolian deposits on Mars also contain some small-scale cross-laminations [59], the lack of larger structures indicative of aeolian environments supports the interpretation that these are of shallow marine origin. In addition, since Mars has only two small moons, it should have low-energy tidal systems, which is what we observe. The bed forms and sedimentary structures described above preserve several features that uniquely identify them as water-laid deposits and not aeolian wind deposits (Supplementary Fig. S9 and Supplementary Table S1). These distinctive features include: (i) the small scale of these deposits contrasts with typical aeolian deposits, which can have much larger-scale cross-laminations; (ii) they are cut by channels that form in subaqueous settings and not in subaerial environments; (iii) the cross-laminations include planar and non-planar types that merge asymptotically with underlying bedding planes; (iv) herringbone cross-strata indicate an alternating or bidirectional depositing current and trough bedding is generally attributed to deposition by variable subaqueous currents; (v) although rare in the observed boulders/blocks, finer-grained or ‘mud-sized’ drapes and flaser-lenticular bedding and occasional candidate larger clasts within finer layers are typical of tidal deposits, with alternating strengths and directions of currents [57].

**Figure 4.**
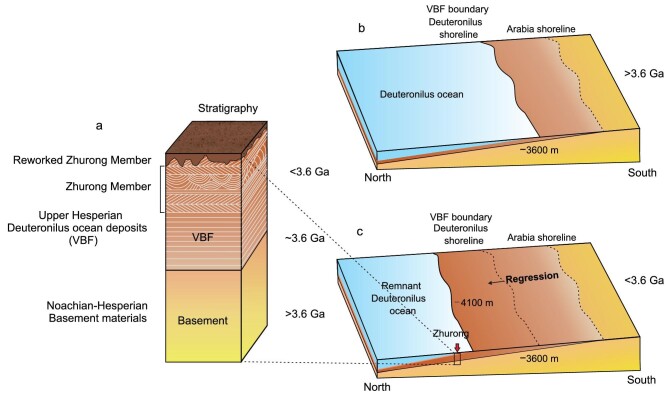
A conceptual model for the Hesperian Deuteronilus ocean, its regression and formation of the oceanic deposits of the Zhurong Member of VBF at the Zhurong landing site. (a) Schematic stratigraphic column of the Zhurong landing site in Utopia Planitia and proximity to the interpreted shoreline. The –3600 m contour in (b) and (c) represents the boundary of the Vastitas Borealis Formation (VBF), a smooth lowland unit consisting of sedimentary deposits of a possible ancient oceanic water body before 3.6 Ga [18,20]. After that, ocean regression may have resulted in a shallow marine environment near the current position of the Zhurong rover (c), which is indicated by the sedimentary structures of the Zhurong Member rocks shown in Figs 2 and 3 and Supplementary Figs S6–S8.

Considering that the landing site is ∼280 km away from and ∼500 m below the proposed Deuteronilus level (Fig. 4), we suggest that the deposition of these sedimentary rocks occurred during the regressional period of a Hesperian ocean. This regressive coastal topography is also present in Chryse Planitia, significantly strengthening our interpretation [24,25]. Indeed, recent ground-penetrating radar results from the Zhurong rover describe the presence of a subsurface multi-layered structure [60] interpreted to have formed by episodic hydraulic flooding sedimentation related to Late-Hesperian flooding and filling of the Utopia basin. In addition, two impact cratering events into such an ocean [22–25], could have formed the megatsunami deposit. The younger of these deposits (but not the older, which lacks evidence of significant backwash [23]) likely involved bidirectional currents, as recently proposed [23,24].

Analysis of the physical character of the sedimentary structures, their groupings [41,46] and comparison with Earth analogs [49,55,58] (Supplementary Fig. S9) strongly suggest deposition in a medium- to low-energy marine environment. These observations provide the most compelling ‘ground’ evidence to date for the marine origin of the Zhurong Member of the VBF and the existence of a Hesperian ocean on Mars. The location of the Zhurong landing site suggests that the observed sedimentary structures could be the result of a regression environment related to the loss of water and demise of a northern ocean (Fig. 4).

## CONCLUSION

We report on the observations of the VBF by the Tianwen-1 Zhurong rover—the first *in situ* observations of a unit interpreted by many to represent the deposits of an ancient Hesperian-aged sea. On the basis of our analysis of layered features documented on rocks imaged and examined by the Zhurong rover, we interpret these to represent sedimentary structures typical of formation in a marine environment, thus providing supporting evidence for the hypothesis that the VBF is a remnant of a Hesperian ocean.

## Supplementary Material

nwad137_Supplemental_File

## Data Availability

The Tianwen-1 data including the Mars Rover Navigation Terrain Camera (NaTeCam), Multispectral Spectral Camera (MSCam) and Micro-imaging Camera data used in this study are processed and produced by the GRAS of China's Lunar and Planetary Exploration Programme and provided by CNSA at https://clpds.bao.ac.cn/web/enmanager/mars1. All the original data are provided with this paper. All the data for this study are accessible at https://doi.org/10.5281/zenodo.7189497. HiRISE data are available from https://www.uahirise.org/ with IDs. MOLA data can also be accessed from https://ode.rsl.wustl.edu/mars/indexProductSearch.aspx. Other data sets generated and analysed in this study are available from the corresponding author upon reasonable request.
