# Supplementary material for: Evidence for marine sedimentary rocks in Utopia Planitia: Zhurong rover observations
Source: Natl Sci Rev. 2023 May 18;10(9):nwad137. doi: 10.1093/nsr/nwad137 (PMC10411667; doi:10.1093/nsr/nwad137)
Supplement: nwad137_Supplemental_File [file nwad137_supplemental_file.docx]

# Supplementary Information

**Evidence for marine sedimentary rocks in Utopia Planitia: Zhurong rover observations**

Long Xiao (肖龙)^1,8^**†***, Jun Huang (黄俊)^1,8^**†**, Timothy Kusky^2,6^*, James W. Head^3^, Jiannan Zhao (赵健楠)^4^, Jiang Wang (王江)^1^, Le Wang (王乐)^1^, Wenchao Yu (余文超)^1^, Yutong Shi (史语桐)^1^, Bo Wu (吴波)^5^, Yuqi Qian (钱煜奇)^1^, Qian Huang (黄倩)^7^, Xiao Xiao (肖潇)^1^

^1^State Key Laboratory of Geological Processes and Mineral Resources, Planetary Science Institute, School of Earth Sciences, China University of Geosciences, Wuhan 430074, China

^2^State Key Laboratory of Geological Processes and Mineral Resources, Center for Global Tectonics, School of Earth Sciences, China University of Geosciences, Wuhan 430074, China

^3^Department of Earth, Environmental and Planetary Sciences, Brown University, Providence RI 02912, USA.

^4^Key Laboratory of Geological Survey and Evaluation of Ministry of Education, China University of Geosciences, Wuhan 430074, China

^5^Planetary Remote Sensing Laboratory, Department of Land Surveying and Geo-Informatics, The Hong Kong Polytechnic University, Hong Kong 100872, China.

^6^Badong National Observatory and Research Station for Geohazards, China University of Geosciences, Wuhan 430074, China

^7^Hubei Subsurface Multi-scale Imaging Key Laboratory, Institute of Geophysics and Geomatics, China University of Geosciences, Wuhan 430074, China

^8^Chinese Academy of Sciences Center for Excellence in Comparative Planetology, Hefei 230026China.

† Authors contributed equally

* Corresponding authors: Long Xiao (longxiao@cug.edu.cn), Timothy Kusky ([tkusky@gmail.com](mailto:tkusky@gmail.com))

**This PDF file includes:**

Materials and Methods

Figures S1 to S11

Tables S1

Materials and Methods

**In situ imaging data**

Imaging data of the Navigation and Terrain Cameras (NaTeCam [1]), the Multispectral Camera (MSCam [2]) and the Micro-imaging Camera [3] onboard the Zhurong rover are used in the detailed geomorphological analysis. The NaTeCams are color binocular stereo cameras with complementary metal oxide semiconductor active pixel sensors, which provide support for the guidance, navigation, and control of the rover. The field of view of each NaTeCam is 46.5° × 46.5° and the stereo baseline is 270 mm.

The NaTeCam has a normal imaging distance from 0.5 m to infinity, and is best focused at 1 m [1]. The images acquired by the NaTeCam are 2048 × 2048 pixels, and the spatial resolution is about 1.5 mm/pixel.

The MSCam is a multispectral imaging system with an angle resolution of 0.146 mrad, therefore, it can take images with a resolution of 0.2 mm at a distance of 1.5 meters. It has 8 spectral bands centered at 480 nm, 525 nm, 650 nm, 700 nm, 800 nm, 900 nm, 950 nm and 1000 nm. We generate color composite images using bands with the central wavelength of 650 nm (Red), 525 nm (Green) and 480 nm (Blue). The greenish tints in the images of MScam are artifacts due to saturation of the sensors. Images of MSCam are stretched to minimize the effects of shadows.

The micro-imaging camera acquires high-resolution images of the targets. The micro-imaging camera has a resolution of 0.1 mm/pixel at a distance of 2 m and 0.25 mm/pixel at 5 m, and an image size of 64 × 64 or 1024 × 1024 pixels [3].

**Orbital remote sensing data**

We use Mars Orbiter Laser Altimeter (MOLA [4]) - High-Resolution Stereo Camera (HRSC [5]) blended digital elevation model (DEM [6]; 200 m/pixel) to analyze regional topography characteristics. Images acquired by High-Resolution Imaging Camera (HiRIC, ~2.5 m/pixel [7]) onboard "Tianwen-1" orbiter and High-Resolution Imaging Science Experiment (HiRISE, ~25–30 cm/pixel [8]) are used to perform detailed geomorphological studies.

**Methods**

High-resolution 3D modelling of rocks

Measurement of the 3D morphological parameters of the rocks and features on rocks is based on 3D models generated by photogrammetric processing [9, 10] of the stereo NaTeCam images acquired by the Zhurong rover. The NaTeCam has two identical cameras separated by a rigid base with a length of 270 mm. Each camera has an image size of 2048 pixels ×2048 pixels and a focal length of 13.17 mm. The cameras can acquire stereo panoramic images through rotation and tilting at each station. Photogrammetric processing of the stereo NaTeCam images involves tie point matching, bundle adjustment, dense image matching, and 3D point cloud generation. The approach begins with automatic extraction and matching of tie points on the stereo pair of images and neighboring pairs of images. The matched points are then used in the bundle adjustment of the image block to refine the image orientation parameters and to achieve consistency within an image block. Dense image matching is then carried out to obtain densely matched points, from which 3D point clouds can be generated by photogrammetric space intersection using the refined image orientation parameters. Finally, 3D mesh models of the terrain surface are generated through triangulation of the 3D point clouds, and the NaTeCam images are then projected onto the 3D mesh models for textural mapping. This photogrammetric approach is similar to the previous works used by [11] and [12], of which the latter was used for detailed topographic mapping at the Chang’E-4 landing site on the lunar surface [12]. Figure S11 illustrates the photogrammetric workflow and examples of the generated 3D mesh models of rocks.

The generated 3D models from NaTeCam images are in local coordinate systems centered at each rover location. The textured models have spatial resolutions of ~ 1.5 mm/pixel at close ranges (e.g., < 5 m) to the rover and ~ 1 cm/pixel at a distance of 10 m from the rover. Their geometric accuracies are evaluated by geo-referencing them to the 0.3 m/pixel HiRISE image covering the Zhurong landing region and comparing the size of common features (e.g., sand dunes, large boulders) visible on both the NaTeCam images and the HiRISE image [13]. The results show discrepancies of 0.9–1.9% for features of sizes ranging from 11 m to 40 m, indicating the favorable geometric accuracy of the generated 3D models. The studied rocks in this analysis are all within approximately 5 m range to the rover. Therefore, the generated 3D models enable accurate and detailed measurement of 3D morphological parameters of the rock features.

Figure S1.


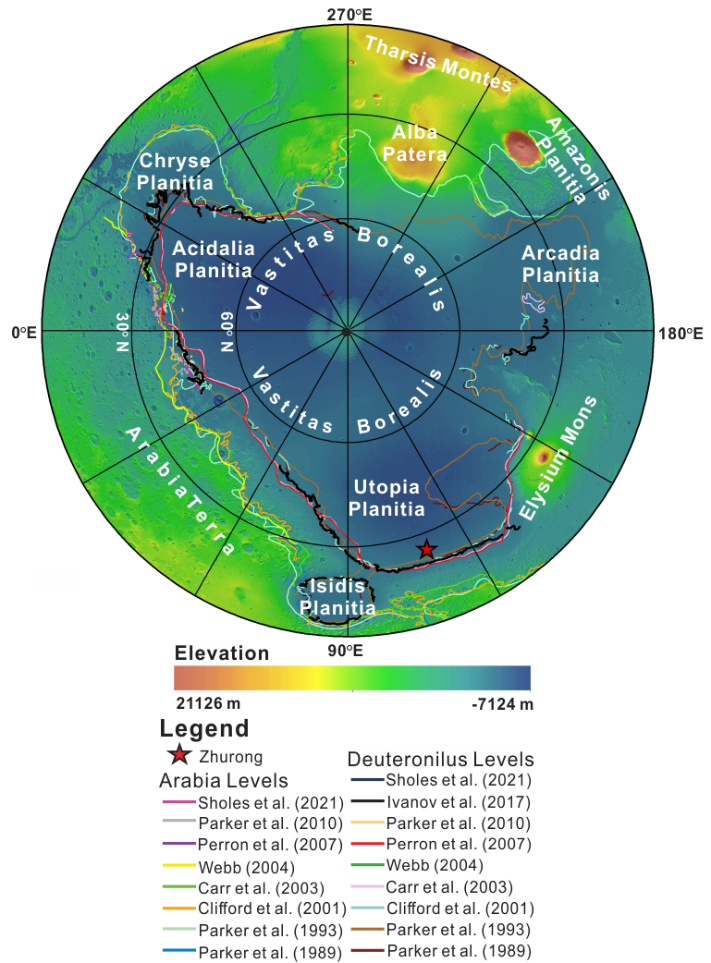


**Figure S1. Topographic map of the northern hemisphere of Mars.** Previously proposed shorelines collected by ref. [14] are shown in solid color lines. The red star denotes the location of the landing site of the Zhurong rover, ~282 km to the north of the Deuteronilus shorelines. The data are color-coded MOLA elevation over MOLA shaded relief centered at the North Pole with the Lambert azimuthal equal-area projection.

Figure S2.

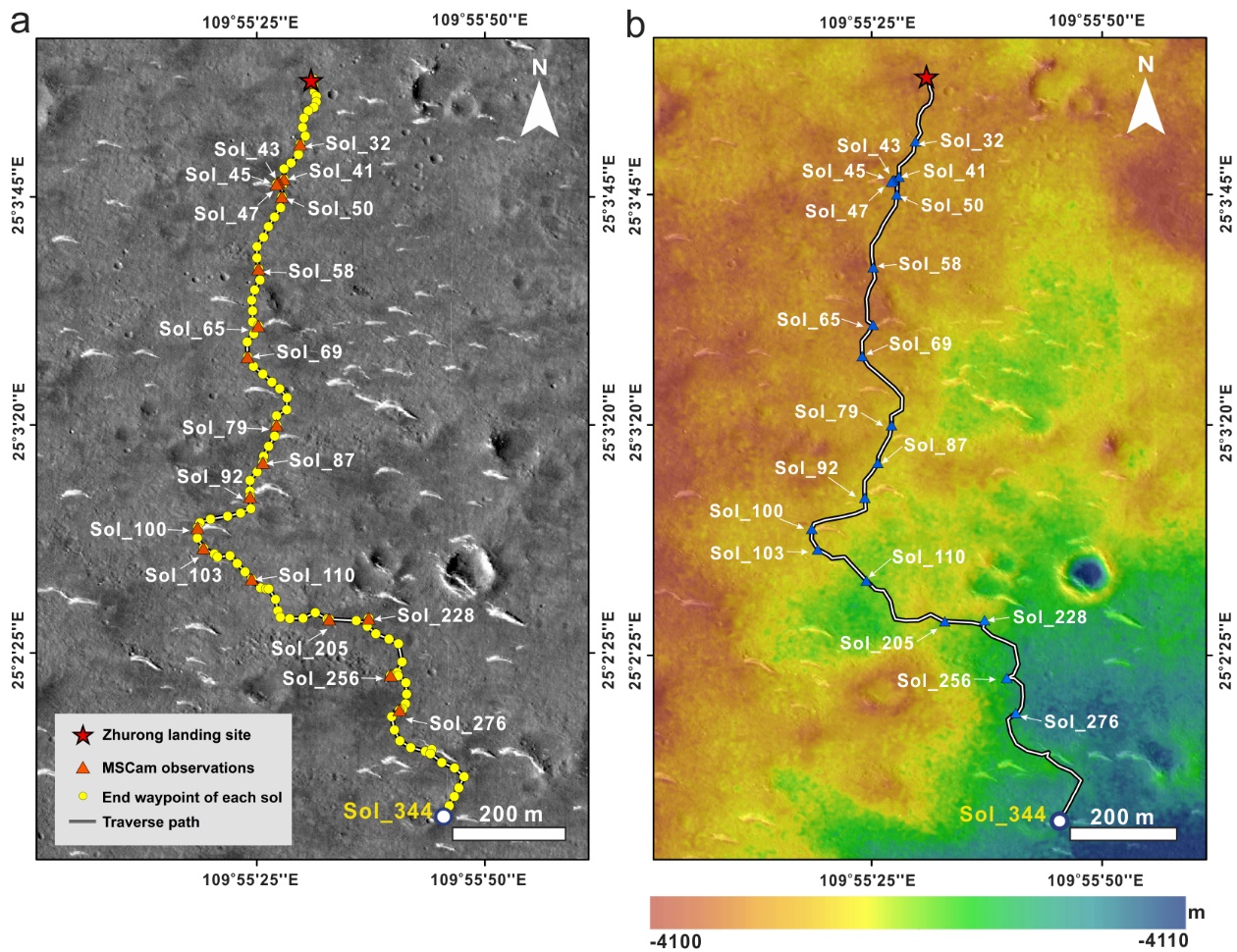


**Figure S2. The ~ 1921-meter traverse of the Zhurong rover as of May 1, 2022 and waypoints for MSCam observations.** The base map of a is HiRIC (HX1_GRAS_HIRIC_DIM_0.7_0004_251515N1095850E_A), b is HiRISE DEM (DTEEC_069665_2055_069731_2055) overlain on the HiRIC. The elevation difference from the landing site to the break point (Sol_344) is about 5 meters.

Figure S3.

**
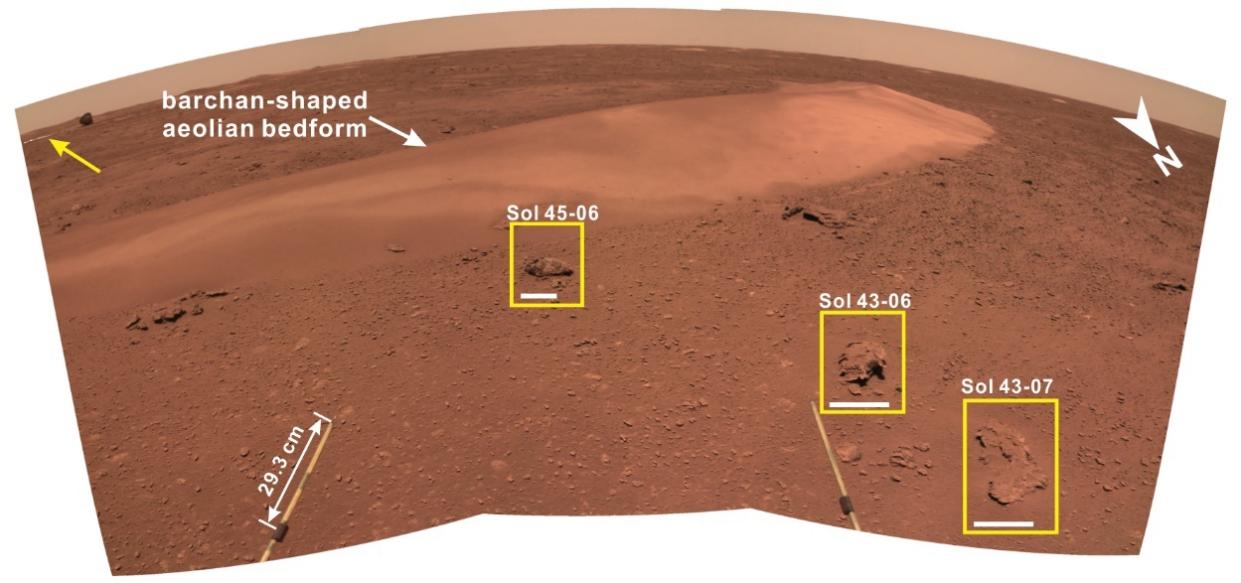
**

**Figure S3**. **NaTeCam panorama acquired in Sol 42.** Blocks Sol 43-06, Sol 43-07 and Sol 45-6 are exposed in front of a barchan-shaped aeolian bedform. The parachute is indicated by the yellow arrow. The scale bars in the yellow rectangles are 15 cm.

Figure S4.


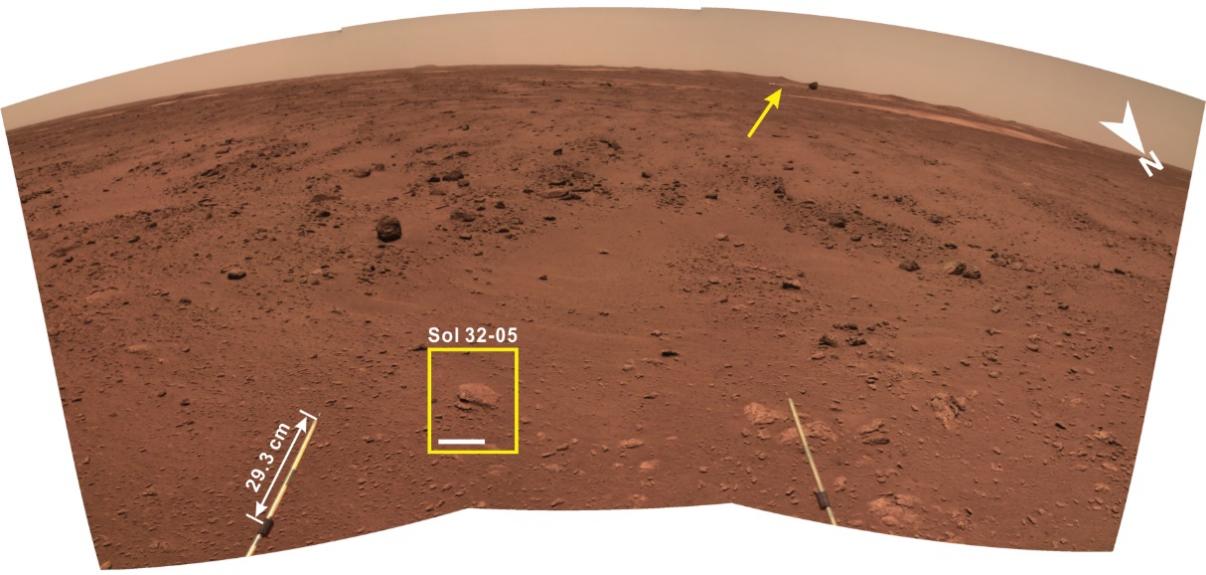


**Figure S4**. **NaTeCam panorama acquired in Sol 31 with an overview of the Sol 32-05 boulder area**., Extensive rocks in various sizes are exposed on the surface. The parachute is indicated by the yellow arrow. The scale bar in the yellow rectangle is 30 cm.

Figure S5.


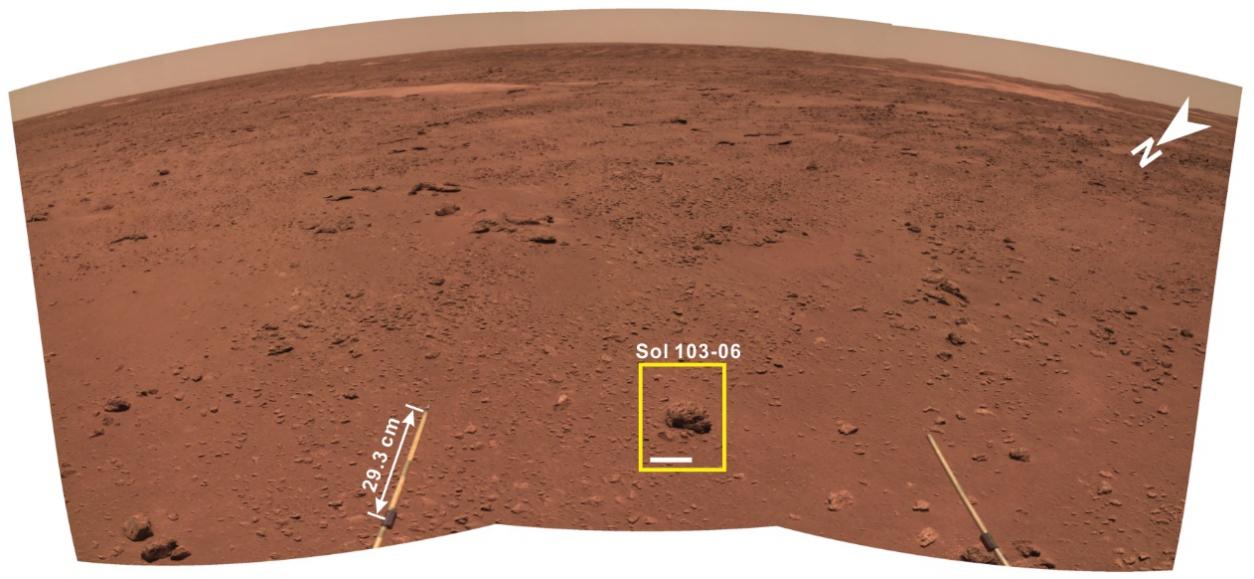


**Figure S5. NaTeCam panorama acquired in Sol 102 with an overview of the Sol 103-06 block site.** Platy and rounded boulders/blocks are seen in the background. The scale bar in the yellow rectangle is 15 cm.

Figure S6.

**
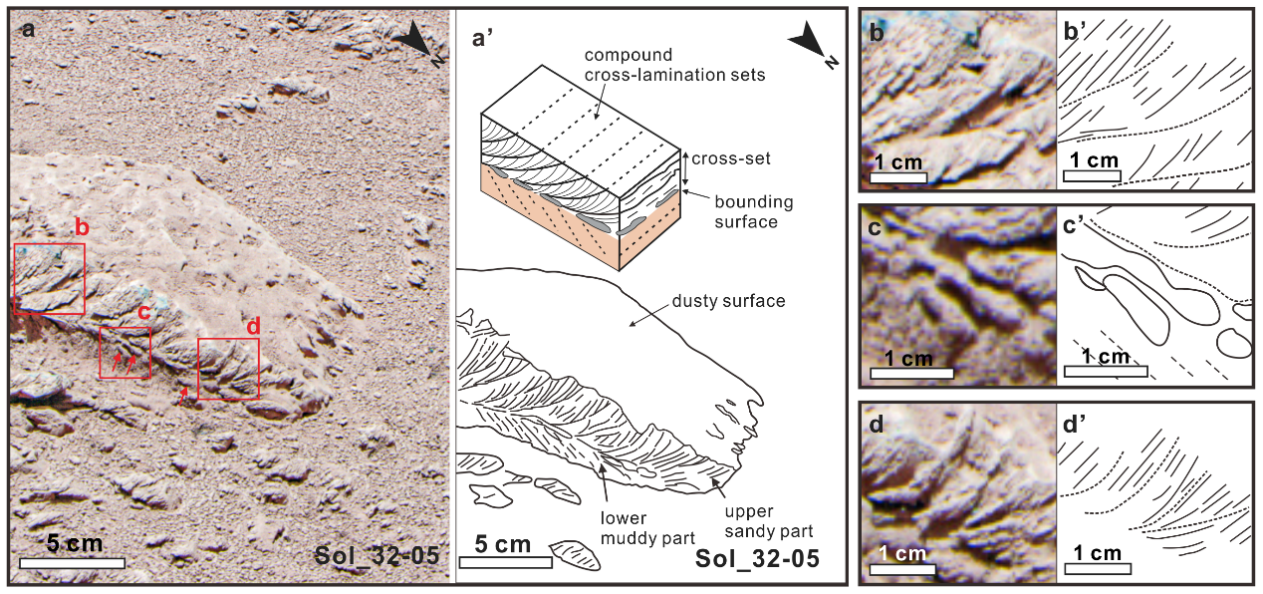
**

**Figure S6. Block Sol 32_05 and sketches of its laminations.** Scene a and a’ show an upper unit of cross-bedded sands forming a coset in a composite bedform, overlying a darker finer-grained unit with faint oppositely-dipping cross-laminae. The boundary between the two units has thin “flasers” of both muddy- and sandy-sized particles. b-d are enlargement and details of sedimentary lamina from image Sol-32_05. b. three tabular sets of trough cross-bedded sands forming a coset in a composite bedform. c. Close-up view of the boundary between the upper trough cross-bedded sand unit, and the lower finer-grained unit with oppositely dipping cross-laminae, with several lenticular sandy horizons preserved along the contact. d. Details of three sets of strongly curved foreset lamina in the upper coset of main cross-laminated unit.

Figure S7.

**
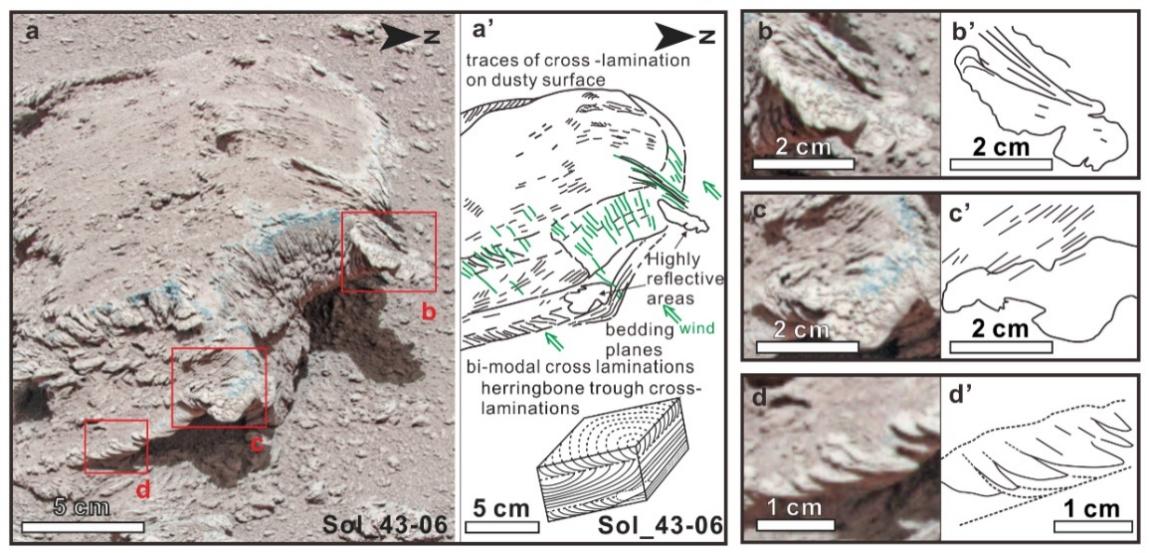
**

**Figure S7. Block Sol 43_06 and sketches of its laminations.** Scene (a, a’) shows several left-dipping beds with internal left- and right-dipping planar and convex cross laminations interpreted to be subaqueous trough cross-beds. The herringbone pattern indicates bi-directional currents. Green lines in a’ show relatively strong wind scars. b and c show relatively large cobble-sized features that are bright, and could be larger clasts in a finer matrix, or surfaces that weathered and are oriented favorably to reflect more light than their surroundings. The detailed sketches show faint lamina within these bright spots that are locally similar to the lamina outside the enigmatic parts of the outcrop, suggesting that it is possible that they are a weathering and lighting phenomena, or similarly-oriented planar-laminated units with different grain size. d shows unusually weathered cuspate trough cross-beds in the lower unit of the outcrop, where the underlying bed has been eroded away, leaving the partially eroded bases of the lamina protruding into the Martian wind.

Figure S8.

**
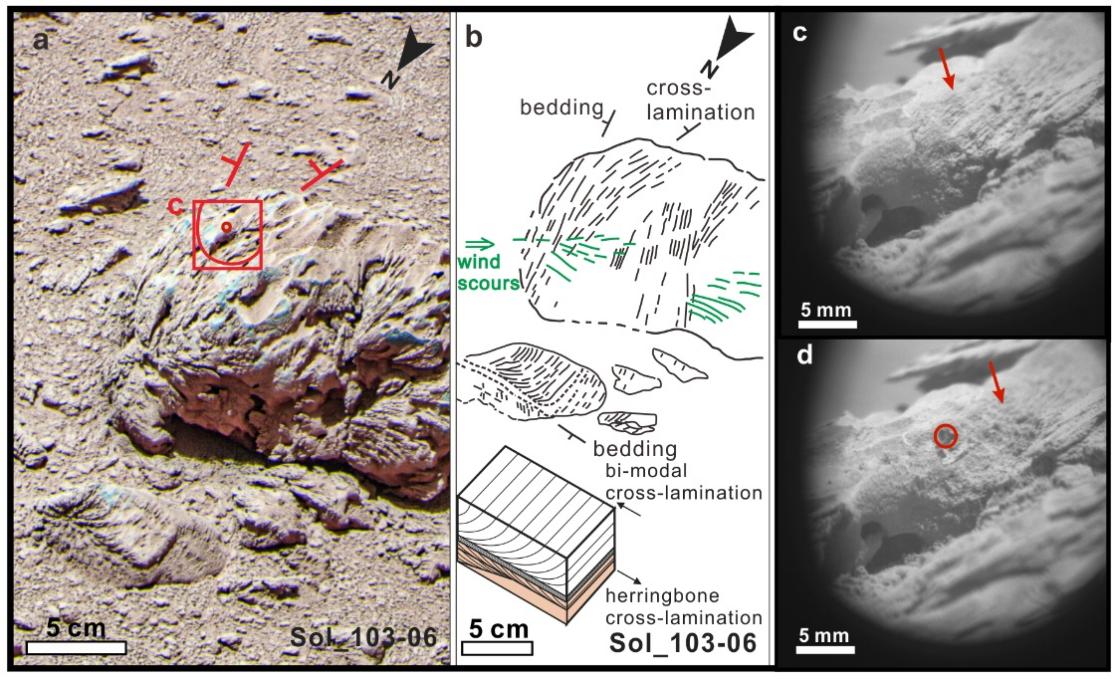
**

**Figure S8. Block Sol 103_06 and sketches of its laminations.** (a) and (b) show photo and line drawings of the main boulder Sol_06 (background) with several ~ 5 cm thick beds of planar and cross-laminated units. Smaller rock in foreground shows convex and herringbone cross-laminations, with flaser-bedded horizons, indicating bi-polar current directions. In panel c and d, the MSCam_Micro System shows the micro-image of the red square area of panel a. Panel c shows the weathered surface of rock, cross-lamina can be observed, panel d is the fresh surface of the rock after the laser-ablation work (ablation spot was highlighted by red circle). In the fresh surface, the cross-lamina still exist (indicated by red arrows), demonstrating that they are the original cross-lamina of the sedimentary rock instead of pseudo-lamina formed by aeolian abrasion or salt crust. This observation lends support to our suggestion in the main text that the wind and other weather factors on Mars has in places enhanced the primary sedimentary features, much as weathering often does on Earth. In panel c and d, comparison between the thickness of lamina-sets and the scale bar indicate the thickness of the lamina-sets is ~5 mm, there are at least 15 lamina in one lamina-set, so the thickness of a single lamina is < 0.3 mm, further estimation of grain size the coarse silt to very fine sand (0.03–0.105 mm).

Figure S9.


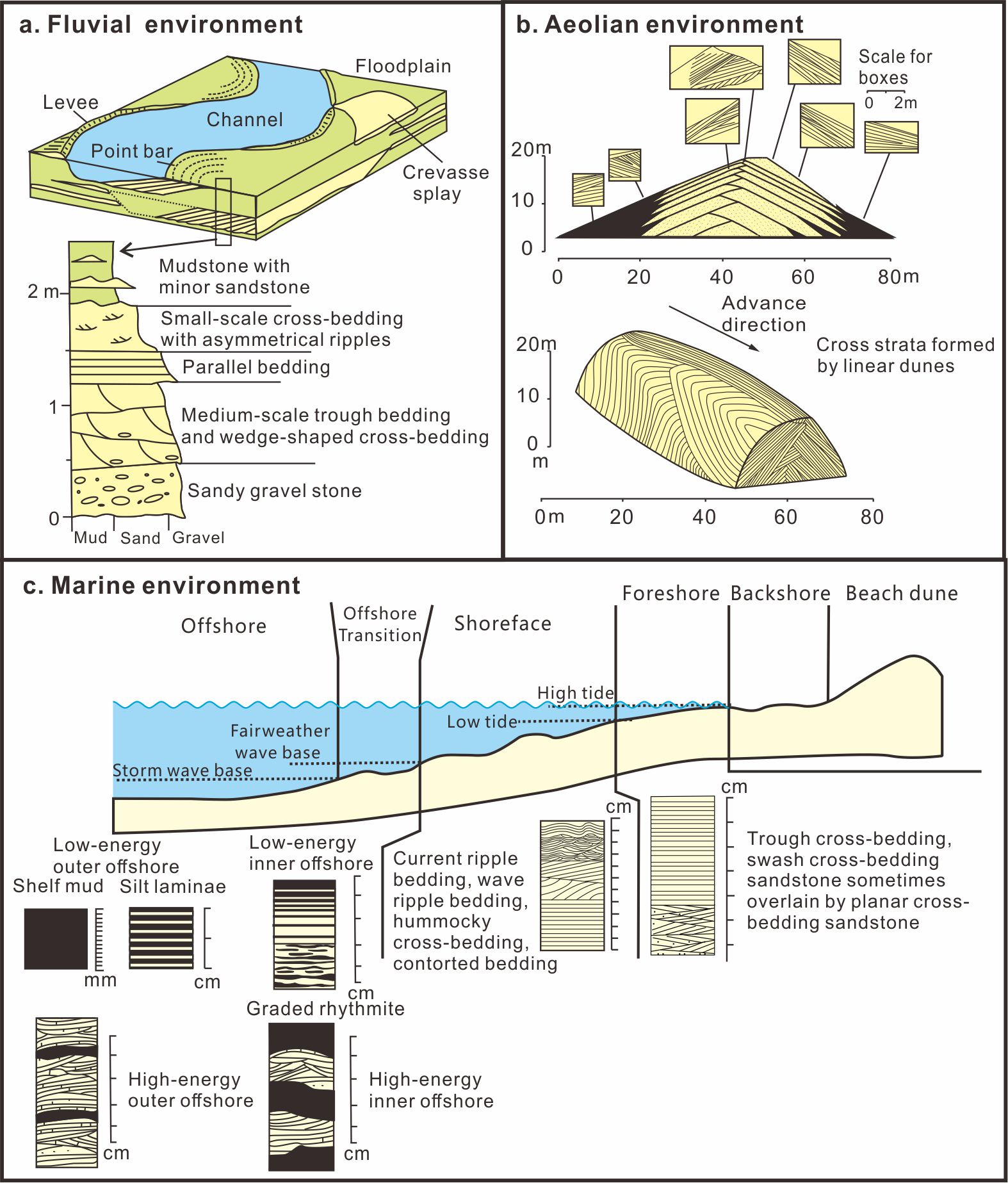


**Figure S9. Summary of representative sedimentary environments and their sedimentary structures on Earth**. (a) Typical fluvial deposit sequence contains a basal sandy gravel layer, middle sandstone layers with medium-scale trough bedding, wedge-shaped cross-bedding, and parallel bedding, upper fine sandstone and mudstone layers [15]. (b) Typical sedimentary structures in aeolian linear dunes, large-scale. Note the different lamination pattern in different parts of the dune. Particularly, trough cross bedding may form large-scale cross strata dipping away from the crest in opposite directions, therefore having a bimodal dip pattern [16]. Martian aeolian dunes are similar with the terrestrial analogue [15]. (c) Typical sedimentary structures in marine environment. Bidirectional cross bedding + though cross bedding + planar cross bedding in the foreshore, current/wave ripple bedding, hummocky cross-bedding and contorted bedding and in the shoreface, and increased mudstone from shoreface to offshore in low-energy condition, but in the high-energy conditions (e.g., storm and tsunami), tempestites turbidites and tsunamiites can be formed. Modified from [17] and SEPM Stratigraphy Web ([http://www.sepmstrata.org/Terminology.aspx?id=shoreline,#](http://www.sepmstrata.org/Terminology.aspx?id=shoreline,)).

Figure S10.

**
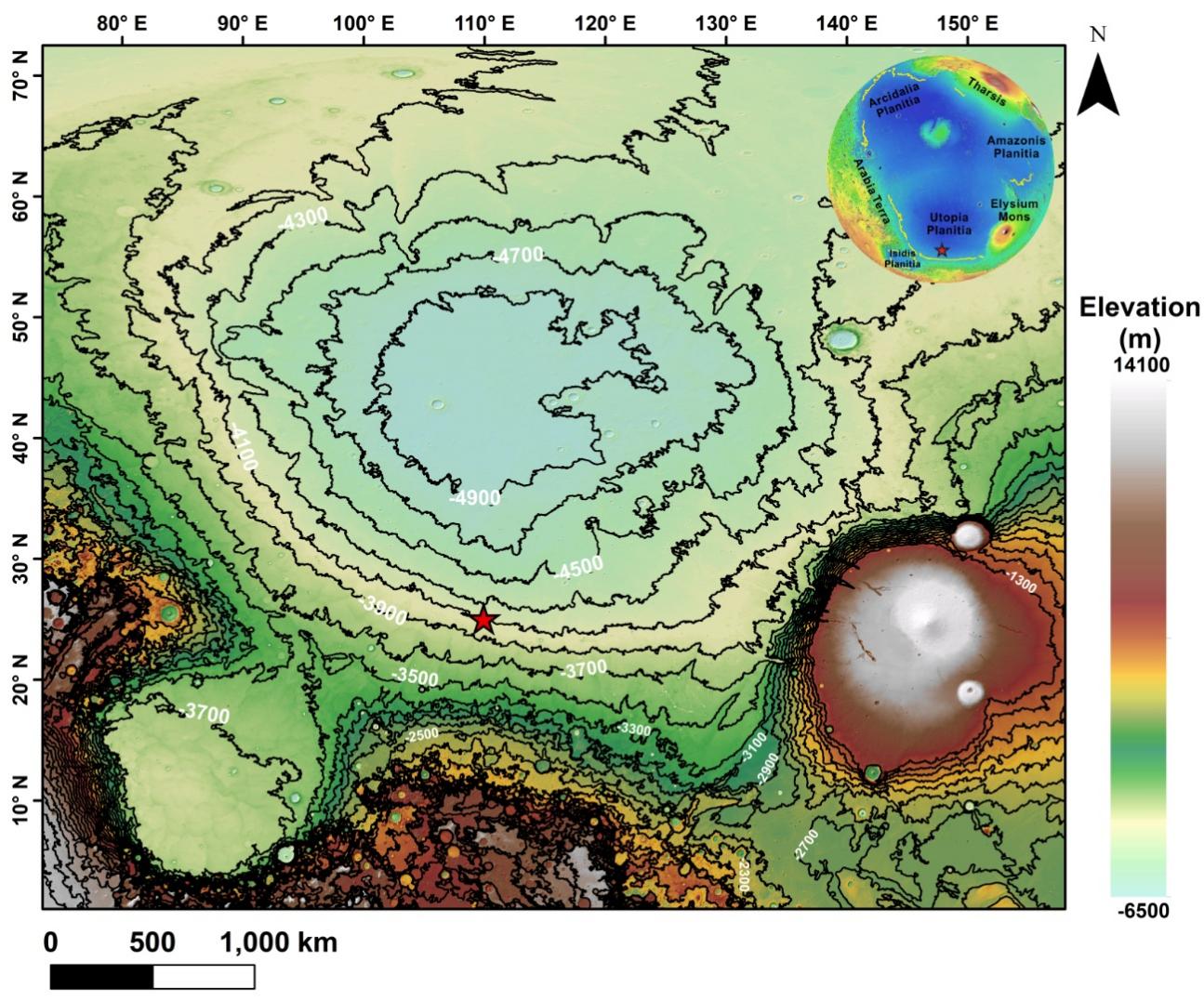
**

**Figure S10. Regional topography with contours of 200-meter intervals showing the Utopia depression and Zhurong landing site (red star).** The Deuteronilus shoreline [18] is indicated by yellow lines in the inset. Zhurong rover is located within the large topography low, the Utopia depression. The data are color-coded MOLA elevation over MOLA shaded relief with the simple cylindrical projection.

Figure S11.

**
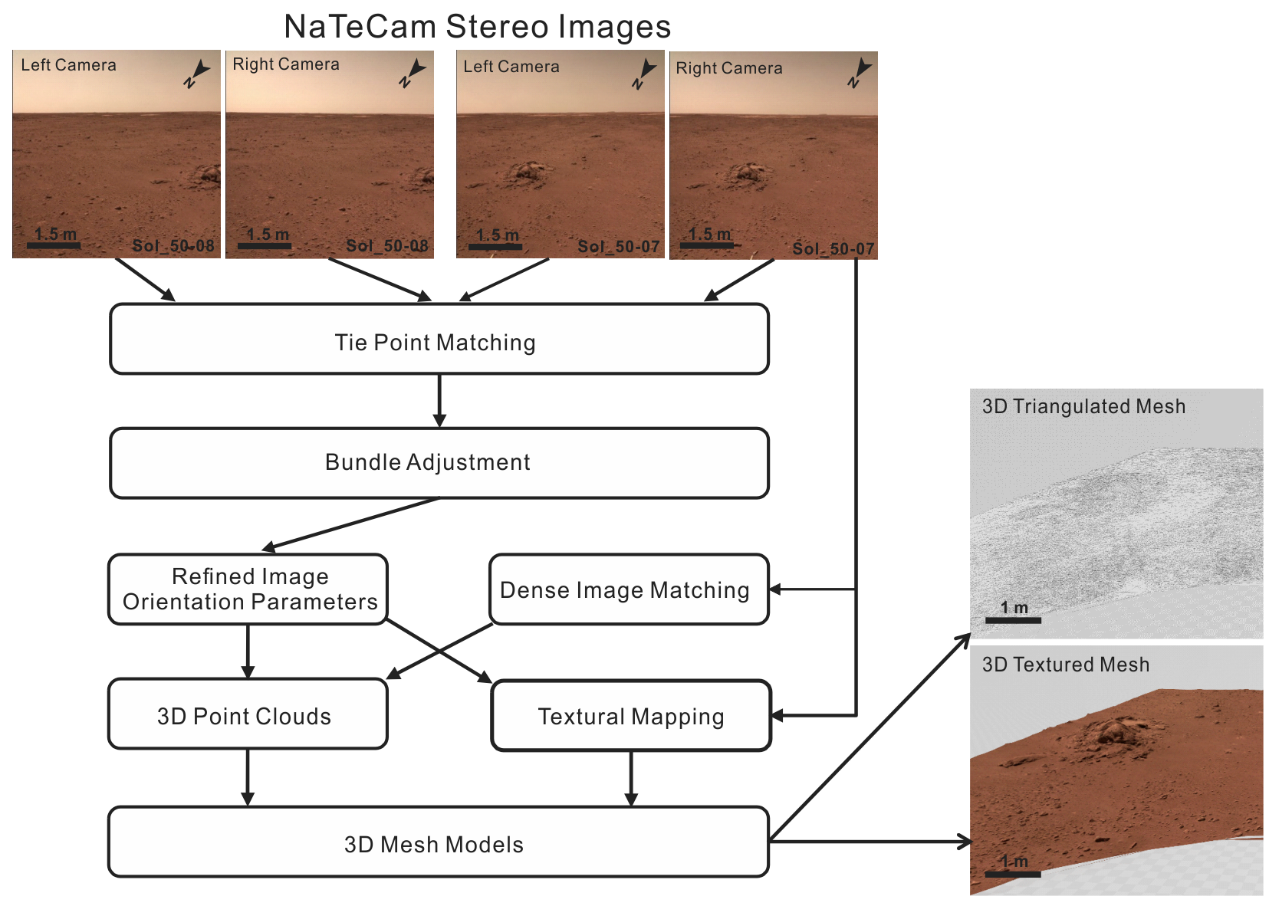
**

**Figure S11. Illustration of the photogrammetric processing to generate 3D mesh models of rocks for measuring morphological parameters.**

**Table S1. Summary of sedimentary facies in this study.** Compared with typical sedimentary structures in Figure S9, assembly of sedimentary structures indicate the foreshore and shoreface facies in the clastic shoreline environment. Grain size estimates were aided by images from the Micro-Imaging Camera (e.g., Figure S8).

| **Facies Table** | | | | | | | | |
| --- | --- | --- | --- | --- | --- | --- | --- | --- |
| **Location** | **Rover** | **Outcrop**  **No./Reference** | **Lithology** | **Speculative particle size** | **Thickness of bedding/laminae** | **Sedimentary structure** | **Facies** | **Deposition environment** |
| Southern Utopia Planitia | Zhurong | Sol_50-8 | Sandstone | Fine-medium sand (0.0625 - 0.5 mm) | Thin (3 - 10 cm) | Parallel lamina; herringbone cross-lamina; trough cross-lamina | Foreshore | Marine |
|  |  | Sol_32-05 | Sandstone and sandy mudstone | Fine sand (0.0625 - 0.25 mm) to silt-mud (<0.0625 mm) | Thin (~5 cm) | Compound herringbone cross-lamina (trough cross-laminated fine sandstone in upper part and tabular cross-laminated sandy mudstone in lower part), lenticular and flaser-type bedding and mud drapes. | Foreshore and upper shoreface |  |
|  |  | Sol_43-06 | Sandstone | Fine-medium sand (0.0625 - 0.5 mm) | Thin (~3 cm) | Trough cross-lamina | Foreshore and upper shoreface |  |
|  |  | Sol_103-06 | Sandstone | Coarse silt to very fine sand (0.03-0.105 mm) | Thin (3 - 10 cm) | Herringbone cross-lamina; internal parallel to slightly oblique laminations; intervening layers show convex cross-laminations | Foreshore |  |
|  |  | Sol_43-07 | Sandstone | Fine-medium sand (0.0625 - 0.5 mm) | Thin (3 - 5 cm) | Parallel lamina, trough cross-lamina, bi-directional cross-bedding, erosion surface, channel | Foreshore and upper shoreface |  |
| Gale crater | Opportunity, Curiosity | ref. (*28*);  ref. (*55*) | Sandstone and siltstone | Medium sand to coarse silt (~0.05 to 0.35 mm) | Thick-massive (0.5 - 1.5 m) | Large-scale cross-beds | Eolian dune | Groundwater-influenced wet aeolian system |
|  |  |  |  |  |  | Planar-laminated sandstone and low-angle cross-stratified sandstone, with intercalated but relatively uncommon intervals of cross-bedded sandstone and rare cross-laminated sandstone | Eolian sand sheet |  |
|  |  |  |  |  |  | Subaqueous current and wave ripples, wavy laminae (wet depositional interface), adhesion structures, desiccation cracks, and wind-ripple stratification (damp to dry depositional interface) | Mixed eolian sand sheet and interdune |  |
| Gale crater | Curiosity | Ref. (*27*) | Mudstone-sandstone | Mud to sand | Thin to thick | Event beds (including scour and drape structures), rhythmically laminated and uneven, discontinously laminated subfacies; low-angle geometric discordances | plunging plumes in a distal lacustrine setting | Fluvial-deltaic system |
|  |  |  | Mudstone | Mud | Thin | variably dipping low-angle cross-stratiﬁcation, froude supercritical bars or bedforms | Sublacustrine channel in a basin margin lacustrine setting |  |
|  |  |  | Mudstone-sandstone | Mud to sand | Thick | Event beds | plunging plumes in a proximal lacustrine setting |  |
|  |  |  | Sandstone | Coarse silt to coarse  sand within ﬁne-grained matrix | Thick | Climbing dune-scale and ripple-scale bedforms | Sublacustrine channel in a proximal basin margin setting |  |

# Supplementary References

1. Liang X, Chen WL and Cao ZX *et al.* The navigation and terrain cameras on the Tianwen-1 Mars rover. *Space Science Reviews*. 2021; **217**(3): 1-20.

2. Yang JF, Liu DW and Xue B *et al.* Design and Ground Verification for Multispectral Camera on the Mars Tianwen-1 Rover. *Space Science Reviews*. 2022; **218**(3): 1-27.

3. Xu WM, Liu XF and Yan ZX *et al.* The MarSCoDe instrument suite on the Mars Rover of China’s Tianwen-1 mission. *Space Science Reviews*. 2021; **217**(5): 1-58.

4. Smith DE, Zuber MT and Frey HV *et al.* Mars Orbiter Laser Altimeter: Experiment summary after the first year of global mapping of Mars. *Journal of Geophysical Research: Planets*. 2001; **106**(E10): 23689-23722.

5. Neukum G and Jaumann R. HRSC: The high resolution stereo camera of Mars Express. In: *Mars Express: The Scientific Payload,* *2004*, p. 17-35.

6. Fergason R, Hare T and Laura J. HRSC and MOLA blended digital elevation model at 200m v2, astrogeology PDS annex. *US Geological Survey http://bit ly/HRSC_MOLA_Blend_v0*. 2018.

7. Meng QY, Wang D and Wang XD *et al.* High resolution imaging camera (HiRIC) on China’s first Mars exploration Tianwen-1 Mission. *Space Science Reviews*. 2021; **217**(3): 1-29.

8. McEwen AS, Eliason EM and Bergstrom JW *et al.* Mars reconnaissance orbiter's high resolution imaging science experiment (HiRISE). *Journal of Geophysical Research: Planets*. 2007; **112**(E5).

9. Ullman S. The interpretation of structure from motion. *Proceedings of the Royal Society of London Series B Biological Sciences*. 1979; **203**(1153): 405-426.

10. Tavani S, Granado P and Corradetti A *et al.* Building a virtual outcrop, extracting geological information from it, and sharing the results in Google Earth via OpenPlot and Photoscan: An example from the Khaviz Anticline (Iran). *Computers & Geosciences*. 2014; **63**: 44-53.

11. Caravaca G, Le Mouélic S and Mangold N *et al.* 3D digital outcrop model reconstruction of the Kimberley outcrop (Gale crater, Mars) and its integration into Virtual Reality for simulated geological analysis. *Planetary and Space Science*. 2020; **182**: 104808.

12. Wu B, Li Y and Liu WC *et al.* Centimeter-resolution topographic modeling and fine-scale analysis of craters and rocks at the Chang'E-4 landing site. *Earth and Planetary Science Letters*. 2021; **553**: 116666.

13. Wu B, Dong J and Wang YR *et al.* Landing Site Selection and Characterization of Tianwen‐1 (Zhurong Rover) on Mars. *Journal of Geophysical Research: Planets*. 2022; **127**(4): e2021JE007137.

14. Sholes SF, Dickeson ZI and Montgomery DR *et al.* Where are Mars’ hypothesized ocean shorelines? Large lateral and topographic offsets between different versions of paleoshoreline maps. *Journal of Geophysical Research: Planets*. 2021; **126**(5): e2020JE006486.

15. Boggs S. Principles of sedimentology and stratigraphy. 2012.

16. Bridge J and Demicco R. Earth surface processes, landforms and sediment deposits. *Earth Surface Processes*. 2008.

17. Nichols G. Sedimentology and stratigraphy. *John Wiley & Sons*. 2009.

18. Ivanov M, Erkeling G and Hiesinger H *et al.* Topography of the Deuteronilus contact on Mars: evidence for an ancient water/mud ocean and long-wavelength topographic readjustments. *Planetary and Space Science*. 2017; **144**: 49-70.
